# On-chip mass spectrometric analysis in non-polar solvents by liquid beam infrared matrix-assisted laser dispersion/ionization

**DOI:** 10.1007/s00216-020-03115-4

**Published:** 2021-01-21

**Authors:** Raphael D. Urban, Tillmann G. Fischer, Ales Charvat, Konstantin Wink, Benjamin Krafft, Stefan Ohla, Kirsten Zeitler, Bernd Abel, Detlev Belder

**Affiliations:** 1 Institut für Analytische Chemie, Leipzig University, Linnéstraße 3, 04103 Leipzig, Germany; 2 Institut für Organische Chemie, Leipzig University, Johannisallee 29, 04103 Leipzig, Germany; 3 Leibniz-Institut für Oberflächenmodifizierung e.V., Abteilung Funktionale Oberflächen, Permoserstr. 15, 04318 Leipzig, Germany

**Keywords:** Microfluidics, IR-MALDI, Non-polar solvents, Reaction monitoring

## Abstract

By the on-chip integration of a droplet generator in front of an emitter tip, droplets of non-polar solvents are generated in a free jet of an aqueous matrix. When an IR laser irradiates this free liquid jet consisting of water as the continuous phase and the non-polar solvent as the dispersed droplet phase, the solutes in the droplets are ionized. This ionization at atmospheric pressure enables the mass spectrometric analysis of non-polar compounds with the aid of a surrounding aqueous matrix that absorbs IR light. This works both for non-polar solvents such as *n*-heptane and for water non-miscible solvents like chloroform. In a proof of concept study, this approach is applied to monitor a photooxidation of *N*-phenyl-1,2,3,4-tetrahydroisoquinoline.

**Supplementary Information:**

The online version contains supplementary material available at 10.1007/s00216-020-03115-4.

**Figure:**
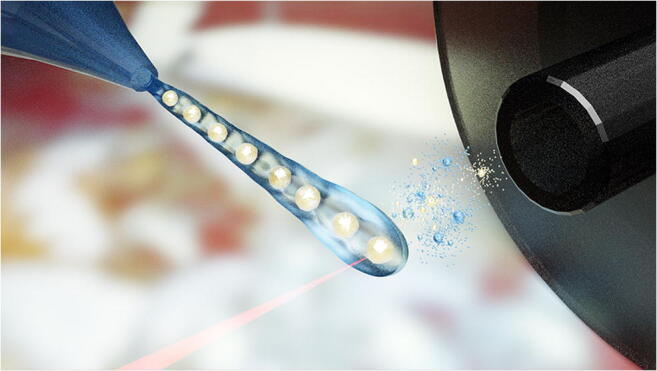
By using water as an infrared absorbing matrix, analytes, dissolved in non-polar solvents from reactions carried out on a microchip, can be desorbed and ionized for investigation by mass spectrometry.

## Introduction

There has been significant progress in coupling microfluidic chips with mass spectrometry (MS) [1–4]. This technique allows to study microfluidic processes on a chip with unsurpassed information content. The most widely applied technology for chip MS hyphenation is based on electrospray ionization (ESI). ESI can be seamlessly integrated on microfluidic devices by the incorporation of an emitter tip. This chip-ESI/MS technology has been widely applied for coupling various microfluidic applications and corresponding devices to mass spectrometry such as chip electrophoresis [5–7], chip chromatography [8–10], microfluidic reaction devices [11–14], droplet microfluidics [12, 15–19], digital microfluidics [20–22], and paper-based devices [23–26]. Most of these chip applications use water or aqueous mixtures with acetonitrile or methanol as the liquid phase. This fact is beneficial for electrospray ionization, which indeed performs best upon using aqueous mixtures of polar solvents. On the other hand, it is challenging to analyze compounds in non-polar solvents by ESI MS.

To overcome this limited application range, ESI has been the subject of intensive research [27]. Approaches described in the literature include atmospheric pressure chemical ionization (APCI) [28–31], atmospheric pressure photoionization (APPI), solvent-assisted electrospray ionization (SAESI) [32–35], desorption electrospray ionization (DESI) [36–38], easy ambient spray ionization (EASI) [39–43], laser ablation electrospray ionization (LAESI) [44], and low-temperature plasma ionization (LTP) [45]. There are practically no literature reports on direct mass spectrometric on-chip analysis of compounds dissolved in water non-miscible, non-polar solvents. The analysis of compounds in non-polar solvents would in principle be feasible using offline approaches such as MALDI-MS [46–49], but this would require additional off-chip steps and would impede real-time analysis. A technology that would allow for on-chip mass spectrometric analyses in non-polar solvents is a missing part in the lab-on-a-chip toolbox with broad applicability, e.g., in micro reaction technology.

We have recently shown that liquid beam desorption mass spectrometry is an exciting alternative to electrospray ionization for microfluidic chips coupling with mass spectrometry [50]. In this approach, a free-standing liquid jet emerging directly from an integrated chip emitter tip is irradiated with an IR laser, promoting desorption and ionization of dissolved analytes. This mass spectrometric technique called laser-induced liquid beam ionization/desorption mass spectrometry (LILBID-MS) [51] or infrared matrix-assisted laser dispersion/ionization MS (IR-MALDI-MS) [52, 53] allows to desorb and ionize compounds in liquid beams using solvents containing hydroxy groups [54–57]. Compared to ESI, IR-MALDI has higher tolerance towards salts and additives and is even softer towards “big analytes” like biomolecules [58]. IR-MALDI is mainly applied to analyze compounds in aqueous media, as the method utilizes water or alcoholic solvents as an absorbing matrix. The absorption of infrared light due to the OH stretch vibration of the aqueous or alcoholic medium is a prerequisite to promote the crucial generation of gas-phase ions of dissolved analytes [59–64]. While this works well for polar compounds, e.g., for biochemical applications, it severely restricts the application scope in synthetic organic chemistry where non-polar solvents are widely used. So even though liquid beam IR-MALDI is an interesting alternative to ESI for chip/MS coupling, it is subject to similar restrictions concerning applied solvents.

Herein, we report the feasibility of performing chip IR-MALDI with liquid jets to analyze compounds dissolved in non-polar and water non-miscible organic solvents. Since one of the main applications of this technology is seen in the field of flow chemistry and reaction monitoring, it was exemplarily used to study the photochemical synthesis of an iminium ion from *N*-phenyl-1,2,3,4-tetrahydroisoquinoline in chloroform.

## Experimental

### Materials and methods

#### Instrumentation

**Fig. 1:**
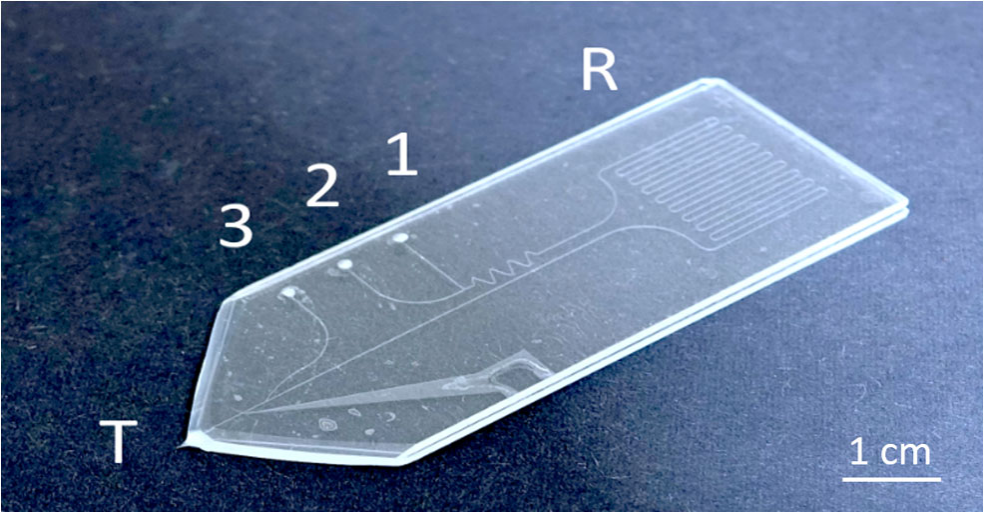
Image of the used microfluidic chip with inlets 1, 2, and 3. R indicates the reactor region and T the emitter tip of the chip

The solvents were delivered by an HPLC piston pump (Merck Hitachi L6200, Merck KGaA, Darmstadt, HE, DEU), a NEMESYS high-pressure pump, and/or two NEMESYS low-pressure syringe pumps (CETONI GmbH, Korbußen, TH, DEU) equipped with two 100-μL glass syringes (Hamilton Bonaduz AG, Bonaduz, GR, CHE). The syringes and the microchip were interconnected by fused silica capillaries (360 μm outer diameter (OD), 75 μm inner diameter (ID); CS-Chromatographie Service GmbH, Langerwehe, NW, DEU), the chip (Fig. 1) was fluidically connected to peripherals via capillaries using steel connection clamps [65].

For precise positioning, the commercial ESI interface of the MS was replaced by an in-house built positioning table with a precision manipulator (OWIS GmbH, Staufen i.Br., BW, DEU). The laser source (Opolette IR 2731, OPOTEK, Carlsbad, CA, USA) was operated at the following settings: wavelength: 2940 nm; frequency: 20 Hz; 3 mJ pulse energy. The chip emitter was positioned at a distance of 2 mm in front and 1 mm above the MS orifice. The chip was aligned so that the liquid beam, MS ion path, and laser beam were on an imaginary orthogonal 3-D coordinate system. The 15-cm-long optical pathway of the laser beam consisted of Ag-coated mirrors (PF10-03-P01) and a CaF_2_ plano-convex lens (*f* = 7.5 cm; Edmund Optics, Barrington, NJ, USA) with a spot size of 0.28 mm^2^. The MS (1100 Series, LC/MSD Trap, Agilent Technologies Inc., City of Santa Clara, CA, USA) was used in ion current control (ICC) active mode, with 4 L/min drying gas, 300 °C gas temperature, *m/z* 100–500 mass range, ion dwell time in the trap: variable from 5 to 100 ms, and spray shield voltage of 0 V.

In order to produce stroboscopic images, the liquid jet was positioned between a light-emitting diode (LED) (KINGSO 5 mm, white) and a self-designed mobile microscope (Thorlabs Inc., Newton, NJ, USA), already described in a previous publication, with a camera connected to it (FLIR Flea 3, FL3-U3-20e4M-C, FLIR Systems, Inc., OR, USA) [8]. The laser beam was aligned orthogonally to the liquid jet. The timing was accomplished as follows: The laser triggered a pulse generator (Stanford Research System DG535, Stanford Research Systems, CA, USA), which, after an adjustable delay, sent a TTL voltage pulse (1 μs) to the LED and camera (see Supplementary Information (ESM) Figs. S4 to S8, pages 6–9). For images without laser bombardment, the timing was set directly by the pulse generator. A description of the stroboscope setup can be found in the ESM, page 6.

#### Microchip design and fabrication

**Fig. 2:**
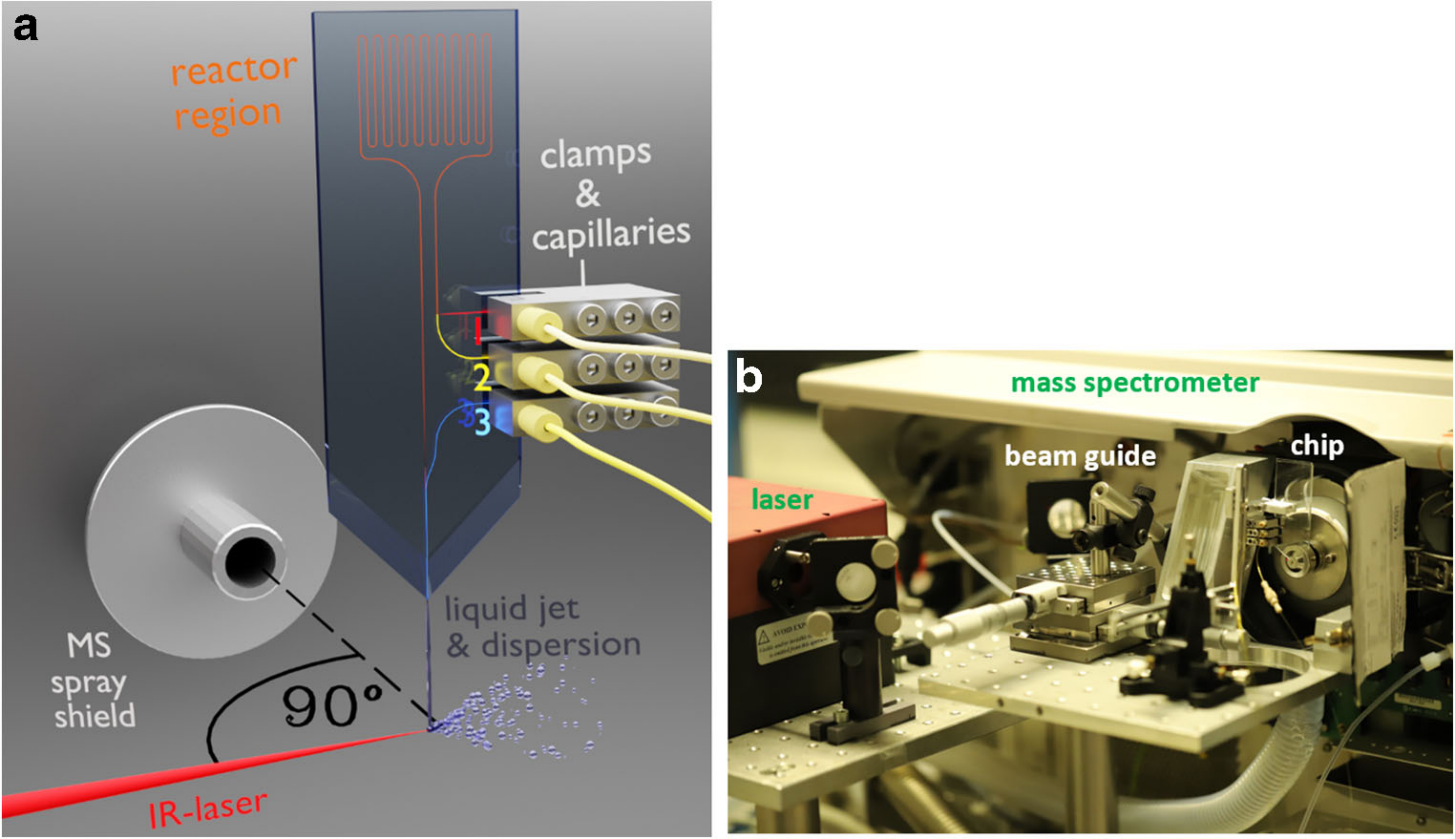
**a** Schematic drawing of the microchip liquid beam desorption mass spectrometry system. A liquid jet is generated at the microchip emitter and irradiated with a pulsed IR laser beam, thereby dispersing the liquid. Analyte solutions are introduced into the channels at inlets 1 (red) and 2 (yellow) using capillaries fixed by high-pressure clamps and mixed in the following channel segment. Water is introduced at the make-up flow inlet 3 (cyan). The MS ion path, the liquid beam, and the laser path were each aligned along the *x*, *y*, and *z* axes. It is indicated that the liquid jet disperses when hit by the laser, and the droplet cloud moves in laser beam direction due to recoil pressure. **b** Image of the setup in front of the mass spectrometer. An in-house built stage positions the chip in front of the mass spectrometer inlet. Mirrors guide the infrared laser beam to a lens that focuses the beam onto the from the chip ejected liquid beam

The microfluidic glass chips, depicted in Fig. 1, were designed and fabricated in-house by standard photolithography, wet etching, and thermal bonding methods [66]. A detailed description is found in the ESM, page 3. Briefly, the chip consisted of two layers. Both layers were manufactured out of microscope slides of soda lime glass with a size of 76 mm × 26 mm (Carl Roth GmbH + Co. KG, Karlsruhe, BW, DEU). The bottom glass layer contained etched channels with a height of 20 μm and a diameter of 80 μm. To close the channels, the slide was thermally bonded to a top layer with powder blasted holes that serve as inlets for pressure-tight fluid delivery. The microchip had the following functional units, as shown in Fig. 2: Two interconnected inlet channels, a meandering region that improves the mixing of liquids, and a third make-up inlet that allows the introduction of water as matrix and make-up flow. An image of the setup is provided in the ESM, Fig. S1, page 4. Flow rates from liquids through inlet 1s and 2 and the make-up inlet will be addressed as *u*_1_, *u*_2_, and *u*_3_ respectively. The reactor’s total length from the intersection of the two channels and the make-up flow channel was 327 mm. The reactor had an internal volume of ~ 520 nL (ESM, Fig. S2, page 4). The outlet of the chip was equipped with an integrated monolithic emitter that narrows the inner diameter of the channel down to 20 μm (ESM, Fig. S3, page 5), which enables the generation of a liquid beam at lower flow rates [67]. A pyramidal-shaped emitter tip was manufactured using a grinding machine. Then, a small conical rod of borosilicate glass was fused to the tip by heat provided by an electrified wire coil of constantan. Afterward, the fused glass joint was heated again, and the emitter tip was pulled out by the gravity of the conical borosilicate glass. The exit of the channel was opened by grinding with superfine microgrit (ISO P2500). The inner diameter of the emitter was approximately 20 μm, and a liquid beam could be established at a flow rate of 80 μL/min water. Unless otherwise specified, the make-up flow was always operated at a flow rate of 120 μL/min. Since the samples form a co-flow or droplets with the water matrix after the reactor-make-up flow channel junction, the sample concentration is always indicated so that it corresponds to the concentration in the reactor channel. Photochemical reactions were irradiated with twelve Osram Oslon SSL royal blue (455 nm) light-emitting diodes (LED, OSRAM GmbH, München, BY, DEU) attached to an aluminum heat sink. The LEDs were operated at 700 mA per LED.

#### Chemicals

All chemicals were used as received. Caffeine, formic acid, Pirkle’s alcohol, hexobarbital, anthracene, and pyrene were purchased from Sigma-Aldrich GmbH (Taufkirchen, BY, DEU). HPLC grade chloroform (CHCl_3_, purity: 99.8%) and *n*-heptane (purity: 97%) were acquired from VWR International GmbH (Darmstadt, HE, DEU). Photoresist AZ 1518, Developer AZ 351B, and chromium etchant AZ® ECI 3000 were bought from Microchemicals GmbH (Ulm, BW, DEU). High-purity water was obtained from a Smart2Pure purifying system (0,055 μS/cm, TKA Wasseraufarbeitungssysteme GmbH, Niederelbert, RP, DEU). Starting materials and product of the model reaction *N*-phenyl-1,2,3,4-tetrahydroisoquinoline were provided by the Zeitler research group (Institute for Organic Chemistry, Leipzig University, Leipzig, SN, DEU) and synthesized as previously reported [68]. The following solutions were prepared: Pirkle’s alcohol, hexobarbital, pyrene in *n*-heptane with the concentrations 0.18 mM, 0.05 mM, 5 mM, and 5.6 mM, respectively. Pyrene, anthracene, *N*-phenyl-1,2,3,4-tetrahydroisoquinoline, and CBrCl_3_ in CHCl_3_ have the concentrations 5 mM, 5.6 mM, 1 mM, and 1 mM respectively. Caffeine in CHCl_3_ have concentrations of 1.5, 3.0, 10, 20, and 50 μM. Pyrene in n-heptane have concentrations of 0.05, 0.25, 0.5, and 1 mM.

## Results and discussion

In initial experiments combining liquid beam IR-MALDI with chip-based droplet microfluidics using water as the continuous phase and water non-miscible solvents as the droplet phase, signals of compounds in the water non-miscible phase were observed unexpectedly. In contrast, no signals were detected without laser irradiation. When solvents without inherent OH groups are irradiated by a laser beam at the wavelength from 2900 to 3100 nm, basically neither desorption nor ionization of dissolved analytes occurs unless plasma discharge appears. To further investigate this finding, we started a comprehensive study. Microfluidic glass chips with a make-up channel were designed in order to dose water to the non-polar and non-miscible solvents just before the liquid jet is formed (Fig. 2 inlet 3).

**Fig. 3:**
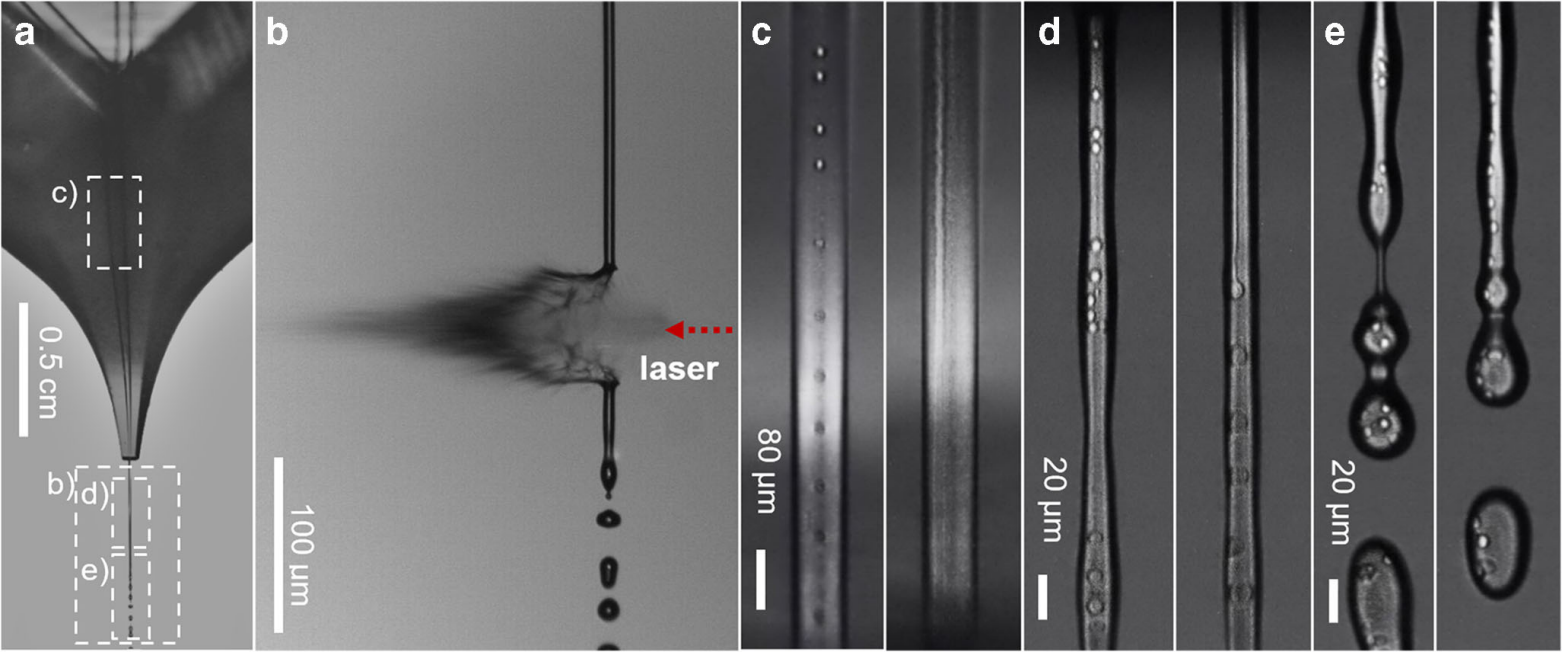
Stroboscopic images of the two-phase liquid beam in air directly after leaving the chip emitter. **a** Schematic image of the chip emitting a liquid jet. Rectangles with a dashed border indicate the position of clippings **b**, **c**, **d**, and **e**. In all images the flow rate u_3_ of water was 120 μL/min. **b** Shows the desorption process 3 μs after laser impact from the right. It can be seen that the liquid jet receives an impulse (recoil pressure) from evaporating water and is pushed in the direction of the laser beam, with the laser beam indicated in red (objective: × 4 magnification). **c**–**e** Stroboscopic images displaying the biphasic system consisting of water and *n*-heptane still in the chip channel, where the flow rate *u*_1_ of *n*-heptane was 5 μL/min on the left and 10 μL/min on the right (objective: × 20 magnification). **c** Liquid beam directly after ejection. **d** Above 10 μL/min, the co-flow decomposition into droplets occurs downstream in the liquid jet. **e** The liquid jet breaks up into biphasic droplets in air. Images of the desorption process at different times after laser exposure are provided in ESM, Fig. S5. An indication of the scale of the liquid jet, size of laser impact, and size of droplets are provided in the ESM, Figs. S6 to S8

We studied the process by video microscopy to visualize the liquid-phase dispersion process as well as the desorption process when the laser hits the liquid beam (Fig. 3). From literature reports, it is known that different flow patterns can be formed when non-polar phases and water are pumped together in microchannels [12, 66, 69, 70]. The outcome of this process is affected by the channel geometries, the ratio of the applied flow rates of the polar and non-polar liquid and the capillary number *C*_a_ = (*η*_c_*v*_c_)/*γ*, where *η*_c_ is the viscosity of the continuous phase, *v*_c_ the average velocity of the continuous phase, and *γ* the interfacial tension between the continuous and dispersed phases. At a low flow rate ratio, droplets are formed in a carrier flow, whereas at a balanced ratio, a co-flow is formed. The decay of the co-flow into droplets shows a hysteresis behavior depending on the history of the applied phase flow rates [71].

In order to visualize these processes in our setup without blur, where relatively high carrier flow rates (hence flow velocities) are required to generate the liquid jet, a stroboscopic imaging setup was used. In a set of experiments, *n*-heptane was pumped through inlet 1 with different flow rates (*u*_1_ = 1, 2, 5, 10, and 20 μL/min) and water was added at inlet 3 as a make-up flow with a flow rate of *u*_*3*_ = 120 μL/min. Exemplary pictures illustrating this process are depicted in Fig. 3c, d, and e. The images indicate that the liquid jet consists of a stable cylindrical part, which breaks up into a stream of droplets (Rayleigh decay) after a certain length that scales linearly with the flow rate. If *n*-heptane is pumped additionally via inlet 1, organic solvent droplets appear in the water jet. These form in the microchip channel at low organic solvent flow rates, up to 5 μL/min. With an increased organic solvent rate, a co-flow of *n*-heptane and water forms and the break-up to organic droplets within water occur further downstream, and above 10 μL/min, it occurs off-chip in the liquid jet. At even higher organic solvent flow rates, the co-flow is stable beyond the collapse of the jet. If this process is now reversed and the flow rate of the organic solvent is reduced, the co-flow remains stable down to flow rates of only 2 μL/min. At lower rates, the droplets are again formed in the chip.

If such a free-standing liquid water jet with a co-flow or dispersed consecutive non-polar droplets is irradiated with the laser, the jet disperses and the liquid matter partly vaporizes as shown in Fig. 3b. Due to the laser spot size, always several droplets (3–7 droplets) were hit with the laser, as can be abstracted from the images in the ESM (Figs. S6 to S8, pages 8–9). By comparing stroboscope images at different times after laser excitation, it is possible to determine the time interval in which the liquid jet is completely replaced by new liquid (at 120 μL/min water every 260 μs). If this is extrapolated with the amount of droplets that appear in the beam when 5 μL/min n-heptane is added (~ 10 droplets), the droplet frequency can be estimated in the order of about 38,000 Hz (for the calculation, see ESM Figs. S9 to S10, page 10).

**Fig. 4:**
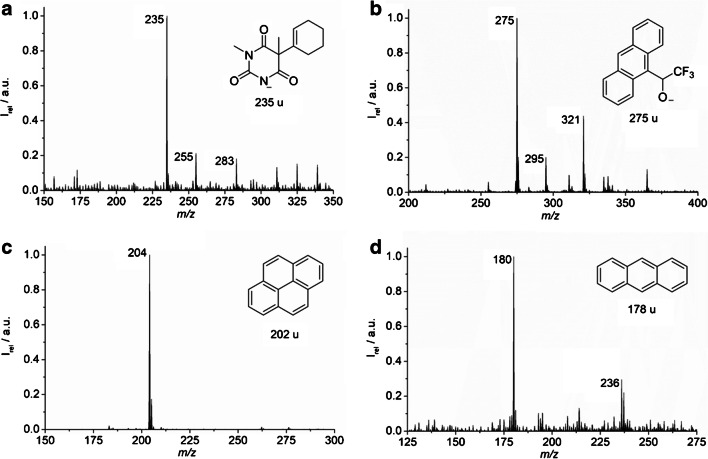
**a** Mass spectra of 0.05 mM hexobarbital and **b** 0.18 mM Pirkle’s alcohol in *n*-heptane in negative ion mode. Given sample concentration is the concentration before mixing with the make-up flow. The signals at *m/z* 235 and 275 correspond to deprotonated anions of the analyte molecules. Spectra of **c** 5.0 mM pyrene and **d** 5.6 mM anthracene in n-heptane in positive mode. The signals of both PAHs are from the species [M+2H]^+●^

To investigate the dispersion and ionization processes for MS compatibility, the microfluidic chip was positioned in front of the entrance orifice of the MS, and an infrared laser beam at 2940 nm was aligned so that it hits the liquid jet perpendicular to the ion path, as shown in Fig. 2. Solutions of hexobarbital or Pirkle’s alcohol in *n*-heptane were pumped via chip inlet 1 at a flow rate *u*_1_ = 5 μL/min. The make-up flow of water occurred at 115 μL/min. The resulting ions after laser irradiation were analyzed in negative ion mode. The exposure of the liquid jet to the IR laser immediately resulted in the significant occurrence of analyte ions and corresponding MS data. While hexobarbital gave no signal in positive ion mode, the negative mode mass spectra in Fig. 4 show signals of deprotonated molecules for both compounds. The MS/MS analysis in negative mode of hexobarbital showed no specific product ions. While the experiment was performed at a flow rate ratio where the organic phase in the laser focus is present as droplets in the water jet, the same ions are also observed when irradiating a co-flow liquid jet at a higher organic flow rate. After successfully ionizing these rather polar compounds, we extended these experiments to truly non-polar compounds with very low solubility in the continuous aqueous phase.

For this purpose, the polycyclic aromatic hydrocarbons (PAH) pyrene and anthracene were chosen as model compounds. The compounds were dissolved in *n*-heptane at a concentration of 10 mg/mL and injected into the chip. As a result, distinctive pyrene and anthracene signals could be obtained. Corresponding mass spectra are depicted in Fig. 4. The base peak for pyrene shows up at *m/z* 204 ([M+2H]^+●^), and for anthracene, the signal is at *m/z* 180 ([M+2H]^+●^). Analogous results were obtained with CHCl_3_ as the solvent; the corresponding mass spectra are provided in the ESM, Fig. S11. The somewhat unusual quasimolecular ions at [M+2H]^+●^ have also been reported earlier for PAH using low-temperature plasma ionization as another soft ambient ionization technique [72]. It was assumed that both react in a Birch-type reaction, which involved electrons from the ion source, and protons from the silicon surface of the interface. While no radical generation from the interface surface is possible in the IR-MALDI setup, quasimolecular ions have been previously reported [73]. Known models for the mechanism of the ionization process are the supercritical phase expansion and incomplete ion recombination model and the liquid dispersion model [61, 74, 75]. In the first, it is assumed that a homogeneously and superheated phase becomes supercritical and expands rapidly after excitation resembling a supersonic hot seeded beam, where cations and anions are generated. Depending on the distance, ions closer to each other will recombine, while those with a larger distance can be isolated. In the second model, it is assumed that the liquid filament is dispersed into nano-droplets with excess charge, which is transferred to contained analytes. Since the signals for PAHs appear at [M+2H]^+●^, the analytes presumably react during the ionization process with the solvent or with each other. As the environment in charged microdroplets differs from that of the bulk, reaction rates are accelerated in charged microdroplets due to extreme pH, an increase of concentration of the reagents, an increased surface area, and increased collision frequencies [76, 77]. In our case, two possible hydrogen and electron donors are plausible: donation from another analyte molecule, or from the solvent, i.e., *n*-heptane, CHCl_3_, or water.

**Fig. 5:**
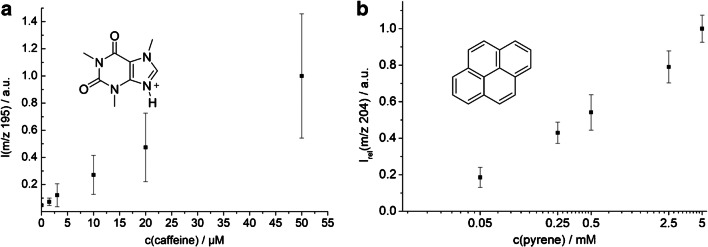
**a** Caffeine 0, 1.5, 3.0, 10, 20, and 50 μM in CHCl_3_ (*n* = 3). **b** Pyrene 0, 0.05, 0.25, 0.5, 2.5, and 5 mM in *n*-heptane (*n* = 5). In both cases, flow rates were *u*_1_ = 5 μL/min and *u*_3_(H_2_O) = 115 μL/min

These preliminary experiments were conducted at relatively high analyte concentrations in *n*-heptane or CHCl_3_, as the matrix “dilutes” the sample depending on flow rates. While these concentrations fit well to typical substrate concentrations in a continuous flow synthesis, the lower concentrations are also worth being investigated. For this purpose, a concentration series of caffeine in CHCl_3_ was measured. The results of the measurement are shown in Fig. 5. The signal of the protonated caffeine scales linearly with the concentration up to about 50 μM. Beyond that, deviation from the linearity starts due to a saturation effect (ESM, Figs. S12 to S13, page 12). A limit of detection of 4.2 μM was determined for this system by linear regression. While certain additives such as formic acid, bases and salts can enhance signals, this depends on the analyte. With caffeine, no significant increase in signal intensity was observed with the addition of formic acid.

The ionization mechanism is still a topic of current research [63]. It would be conceivable that the substances diffuse to some extent from the solvent into the water matrix and are then ionized there. In this case, the signal response of a chemical would hardly increase beyond its solubility in water. To test this, a concentration series of pyrene in *n*-heptane was measured. The solubility of pyrene in *n*-heptane is 12 mM, whereby it dissolves in water only up to a concentration of 0.66 μM [78, 79]. As depicted in Fig. 5, the experiment showed that the signal increases up to a concentration of 5 μM. A comparative measurement was carried out, in which a saturated aqueous pyrene solution was examined. This solution was pumped directly with a high flow rate without adding further water matrix. Therefore, it was not diluted by make-up flow in comparison to the *n*-heptane solutions. In this measurement, the signal of pyrene was hardly distinguishable from noise (ESM, Fig. S11c, page 11). These experiments indicate that most of the pyrene gets ionized directly out of the non-polar phase during the ablation process.

**Scheme 1:**
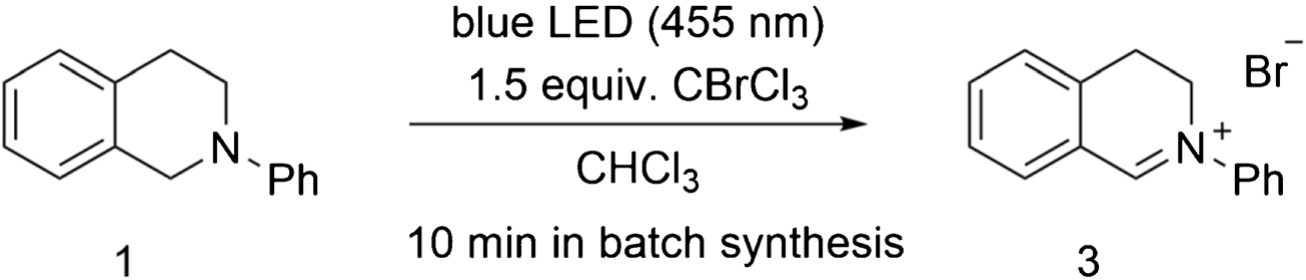
Investigated model reaction: photooxidation of *N*-phenyl-1,2,3,4-tetrahydroisoquinoline (**1**) with CBrCl_3_ (**2**) to *N*-phenyl-3,4-dihydroisoquinolium bromide (**3**) in CHCl_3_

After this first successful realization of on-chip MS detection in water non-miscible solvents, we tested the applicability of this technology for the online analysis of a photochemical organic on-chip reaction. As a proof of concept study, we chose the photooxidation of *N*-phenyl-1,2,3,4-tetrahydroisoquinoline (**1**) (Scheme 1). It is known that the iminium ion *N*-phenyl-3,4-dihydroisoquinolium ion (**3**) can be produced from the tertiary amines tetrahydroisoquinoline by using metal- and photo-catalysis with, e.g., air, oxygen, or CBrCl_3_ as sacrificial electron acceptors [80–82]. Zeitler et al. described that the reaction can be performed with CBrCl_3_ without the use of photocatalysts. These conditions transform isoquinoline **1** to the corresponding iminium ion **3**, while avoiding detrimental interactions with reactive oxygen intermediates [68]. The reaction is known to be very fast, and in batch, a full turnover is reached within minutes.

**Fig. 6:**
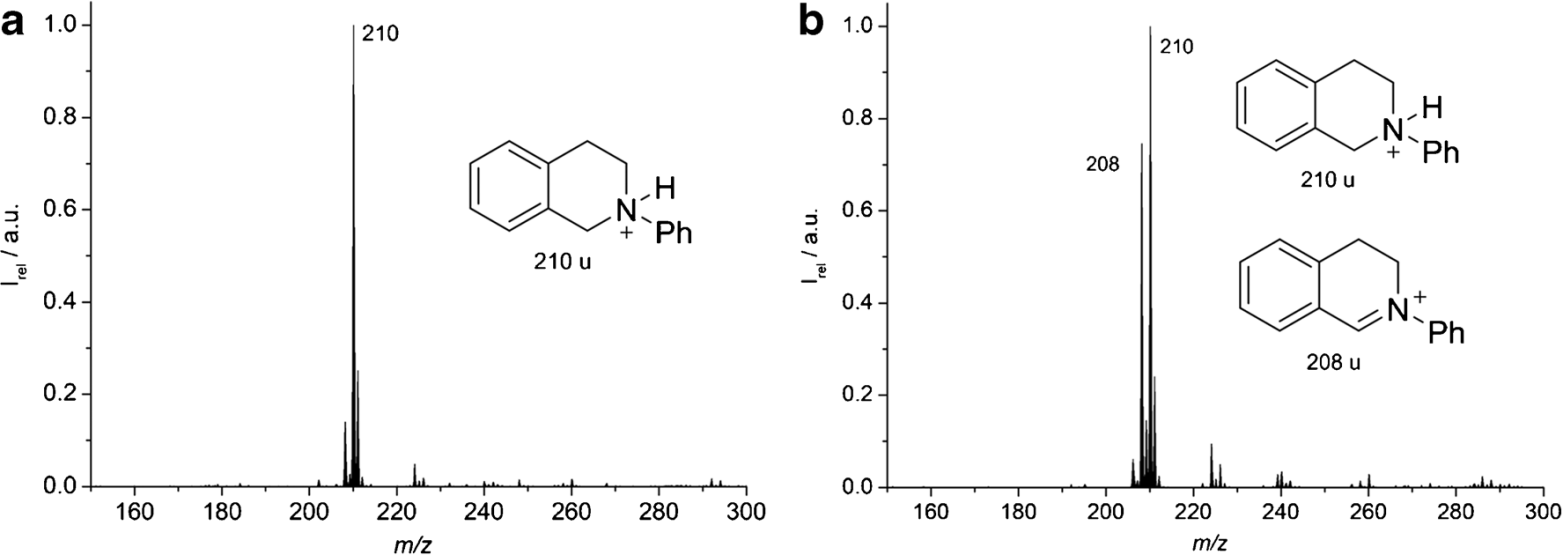
**a** Mass spectrum of **1**. **b** Progress of the *N*-phenyl-1,2,3,4-tetrahydroisoquinoline photooxidation on chip in CHCl_3_ after 16-s residence time, with the corresponding ions of **1** at *m/z* 210 and of **3** at *m/z* 208

To study this reaction on-chip, starting material **1** was dissolved in CHCl_3_ 1.0 mM and pumped at a volume flow rate of 1 μL/min through inlet 1 (NMR spectra for starting materials: ESM, Figs. S14 to S15, pages 14–15). At the end of the reaction channel, water was supplied through inlet 3 at a flow rate of 120 μL/min. The acquired mass spectra of **1** is displayed in Fig. 6a. To start the reaction, 1 mM solution of CBrCl_3_ (**2**) in CHCl_3_ was added at 1 μL/min via inlet 2. This corresponded to a residence time of 16 s of both liquids in the reaction region of the chip (ESM, page 4). To initiate the photoreaction, the chip was illuminated by a blue LED with a wavelength of 455 nm. After adding the solution of compound **2**, the resulting mass spectrum (Fig. 6b) displays the signal at *m/z* 208, which can be assigned to the product of the reaction (iminium ion) **3**. These proof of concept experiments indicate that the presented liquid beam infrared MALDI approach is suitable to monitor a microflow synthesis on-chip occurring in organic solvents.

One limitation of the current setup is that it provides a relatively low signal intensity compared to electrospray ionization. The reasons for this are that the sample is strongly diluted with the aqueous matrix to produce a liquid jet and that only a fraction of the sample is covered by the slow laser frequency. Future work is planned to address these technical aspects more thoroughly, in order to improve the signal intensity.

## Conclusion

In this work, we showed the first example of studying chemical processes in water non-miscible solvents on-chip applying IR-MALDI. We used a microfluidic glass chip with a fine pulled emitter tip to generate a stable aqueous jet that contained droplets of chloroform or *n*-heptane. Water hereby acted as a matrix that absorbs IR laser light, inducing desorption and ionization of dissolved analytes in the organic liquid. The technique was used to monitor a transformation of *N*-phenyl-1,2,3,4-tetrahydroisoquinoline in chloroform. With this approach, it is now possible for the first time to follow on-chip reactions in non-polar and water non-miscible organic solvents by mass spectrometry, with correspondingly versatile potential, not only for flow chemistry applications.

## Supplementary information

ESM 1 (PDF 2200 kb).
