# Supplementary material for: On-chip mass spectrometric analysis in non-polar solvents by liquid beam infrared matrix-assisted laser dispersion/ionization
Source: Anal Bioanal Chem. 2021 Jan 21;413(6):1561–70. doi: 10.1007/s00216-020-03115-4 (PMC7921053; doi:10.1007/s00216-020-03115-4)
Supplement: Supplementary file 1 — (PDF 2200 kb). [file 216_2020_3115_MOESM1_ESM.pdf]

Supplementary Information

On-chip mass spectrometric analysis in non-polar solvents by Liquid Beam Infrared Matrix-Assisted Laser Dispersion/Ionisation

Raphael D. Urban, [a] Tillmann G. Fischer, [b] Ales Charvat, [c] Konstantin Wink, [a] Benjamin Krafft, [a] Stefan Ohla, [a] Kirsten Zeitler, [b] Bernd Abel, [c] and Detlev Belder *[a]

[a.] *Institut für Analytische Chemie, Leipzig University, Linnéstraße 3, 04103 Leipzig, Deutschland.*
[b.] *Institut für Organische Chemie, Leipzig University, Johannisallee 29, 04103 Leipzig, Deutschland.*
[c.] *Leibniz-Institut für Oberflächenmodifizierung e.V., Abteilung Funktionale Oberflächen, Permoserstr. 15, 04318 Leipzig, Deutschland.*

Table of contents

1- Microchip fabrication P3

2- Calculation of reactor volume and reactor residence time P4

Figure S1: Images of the chip-IR-MALDI-MS interface

Figure S2: Drawing of the chip layout and channel dimensions

3- Emitter manufacturing process P5

Figure S3: Images of the pulling process for emitter manufacture

4- Setup for strobe photography and images of desorption process P6

Figure S4: Illustration of strobe setup for images of desorption process

Figure S5: Images of desorption process after laser exposure P7

5- Survey of dimensions in liquid beam P8

Figure S6: Illustration of the size of the liquid jet and desorption process

Figure S7: Determination of length of liquid beam

Figure S8: Shadowgraphy image of the two-phasic liquid beam with indicated scale P9

6-Droplet frequency calculation P10

Figure S9: Comparison of images after laser exposure

Figure S10: Counting of number of droplets in liquid beam

7-IR-MALDI mass spectra of Polyaromatic Hydrocarbons P11

Figure S11: IR-MALDI spectra of anthracene, pyrene and benzo(a)anthracene

8- IR-MALDI mass spectra of caffeine in $CHCl_3$ P12

Figure S12: Caffeine mass spectrum in $CHCl_3$

Figure S13: Calibration of caffeine in $CHCl_3$

9- Photochemical Oxidation of *N*-phenyl-1,2,3,4-tetrahydroisoquinoline P14

Figure S14: ^{1}H NMR spectrum of N-phenyl-1,2,3,4-tetrahydroisoquinoline in $CHCl_3$

Figure S15: ^{13}C NMR spectrum of N-phenyl-1,2,3,4-tetrahydroisoquinoline in $CHCl_3$ P15

References P15

1. Microchip fabrication

All chemicals were purchased from Microchemicals GmbH (Ulm, BW, Germany). Soda-lime microscopic glass slides (76 mm x 26 mm, Carl Roth GmbH + Co. KG, Karlsruhe, BW, DEU) were sputtered with a thin chrome layer (150 nm, FHR Anlagenbau GmbH, Ottendorf-Okrilla, SN, DEU). Foil masks were designed with the desired layout by using the open-source vector graphics editor Inkscape (https://inkscape.org/) and printed by DTP-System-Studio GmbH (Leipzig, SN, DEU). After applying a photoresist (AZ1518) onto the cleaned chrome layers of the microscopic slides by drop.casting and spin coating (Spin 120 PTFE, SPS Europe, Putten, GE, NL; setting: 4000 rpm, 1000 rpm/s, 30s) and covering with the photomask, the slides were exposed to the light (14 mJ/cm², 30s) of a mercury arc lamp (Flutbelichter FB5, SÜSS MicroTec AG, Garching, BY, DEU). The photoresist was developed by a mixture (1:4 v/v) of developer (AZ351B) and water. A patterned chrome layer was obtained by etching the layer *via* a mixture of perchloric acid ($HClO_4$), and ceric ammonium nitrate $(NH_4)_2[Ce(NO_3)_6]$ (TechniEtch Cr01), for 1 minute. Afterwards, the chromium patterned microscopic slide was etched with hydrofluoric acid (BOE 7:1 (HF : NH_4F = 12.5 : 87.5 v/v) for 40 min. After neutralizing the HF by a $[Ca(OH)_2]$ – solution and rinsing with water, the residual photoresist and chrome were removed with acetone and chrome etchant, respectively. Cover slides were manufactured by powder blasting inlet holes into microscopic slides for microfluidic contacting (sandblaster Point 2, Barth Serienapparate, Königsbach-Stein, BW, DEU). They were bonded to the structured slides *via* fusion bonding applying a temperature program (heating ramp to 500 °C in 50 min, maintain Temperature for 15 min, heating to 550 °C within 10 min, maintain temperature for 30 min, heating to reach 620 °C within 15 min, maintain temperature for 180 min, commence cool down to room temperature within 600 min) in a muffle furnace (muffle furnaces P330, Nabertherm GmbH, Lilienthal, NI, DEU).

2. Calculation of reactor volume, reactor residence time and setup images

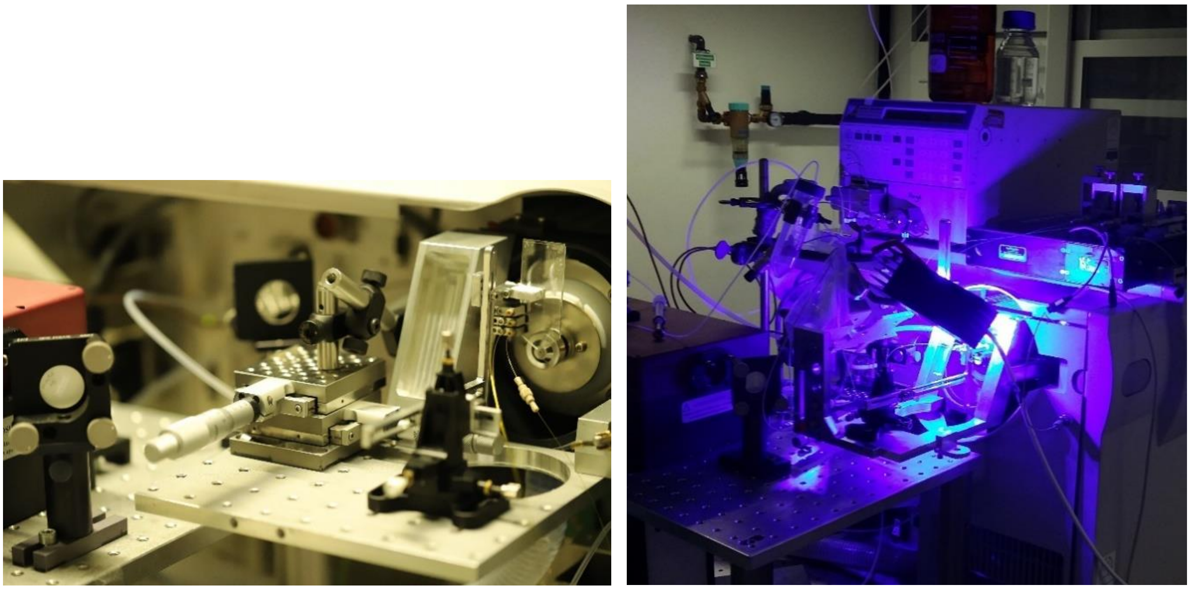

Fig. S1 a) and b) Images of the chip-IR-MALDI-MS interface.

The reactor channel depth of the non-bonded structured glass slide was measured by a Dektak 150 (Veeco Instruments, Plainview, NY, USA) (depicted in Figure S1). As depicted in Figure S1 a), the channel had an average diameter of 80 µm and a height of 20 µm. As calculated in formula 1, the reactor from the junction of inlet 1 and 2 to the makeup flow inlet 3 had an internal volume of 0.523 µL.

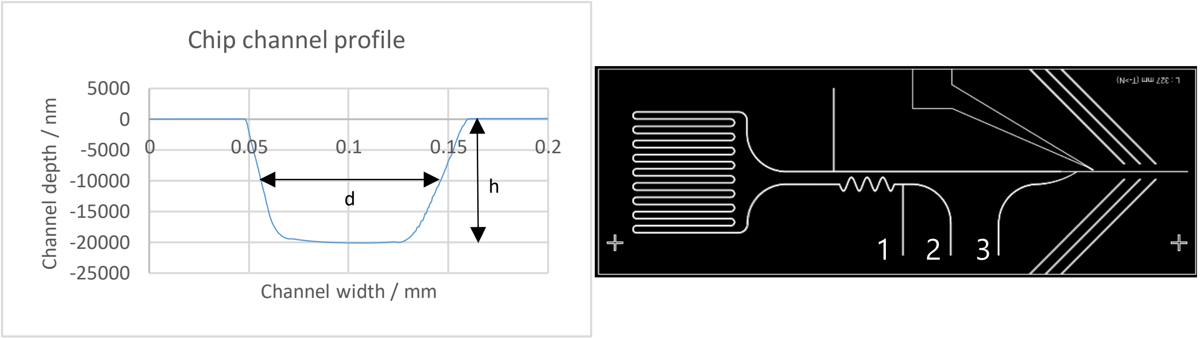

Fig. S2 a) Channel profile of the bottom etched microscopic slide before bonding measured by a surface profilometer. b) Mask of the chip manufacturing process with indicated inlets 1,2 and 3.

$$V(reactor) = h \cdot d \cdot l = 20\ \mu m \cdot 80\ \mu m \cdot 327\ mm = 523nL \approx 0{,}520\ \mu L \quad (1)$$

For the reaction of *N*-phenyl-1,2,3,4-tetrahydroisoqinoline with $CBrCl_3$, the reaction solutions were set to an on-chip flow rate of 1 µL/min *via* inlet 1 and 2, respectively. The on-chip residence time from inlet 1 and 2 to the make-up junction is calculated by division of the reactor volume by the flow rate.

$$t = \frac{V(reactor)}{u_{reactor}} = \frac{523\ \text{nL}}{2\ \mu L/min} \approx 16\ \text{s} \quad (2)$$

With a combined solution flow rate through the reactor of 2 µL/min, the reaction solution stayed in the reactor for approximately 16 s before dilution with water.

3. Emitter manufacturing process

The manufacturing process of an emitter for glass chips with free micro channels was adapted from previous processes [4] and developed according to the schematics depicted in Figure 2, To prepare the chips for the pulling process, the chips were ground to a 45° angle at the desired channel exit, with the channel always facing away from the spinning direction of the sanding sheet (P400 sanding sheet). After dry removal of the glass dust, and rinsing the channels to remove deposited dust, the chip was chucked into a tip pulling device. A glass cone was melted onto the channel exit by pressing it against the ground chip tip, while it was heated by a resistance heated wire (Kanthal A1, d = 1 mm). By moving the heated wire towards the chip, the glass melted and was elongated by gravity force. During the elongation process, the metal wire was moved with the glass cone in a distance 2 mm. The emitter tip was opened by grinding the emitter tip at the desired position by fine abrasive paper (P 2500).

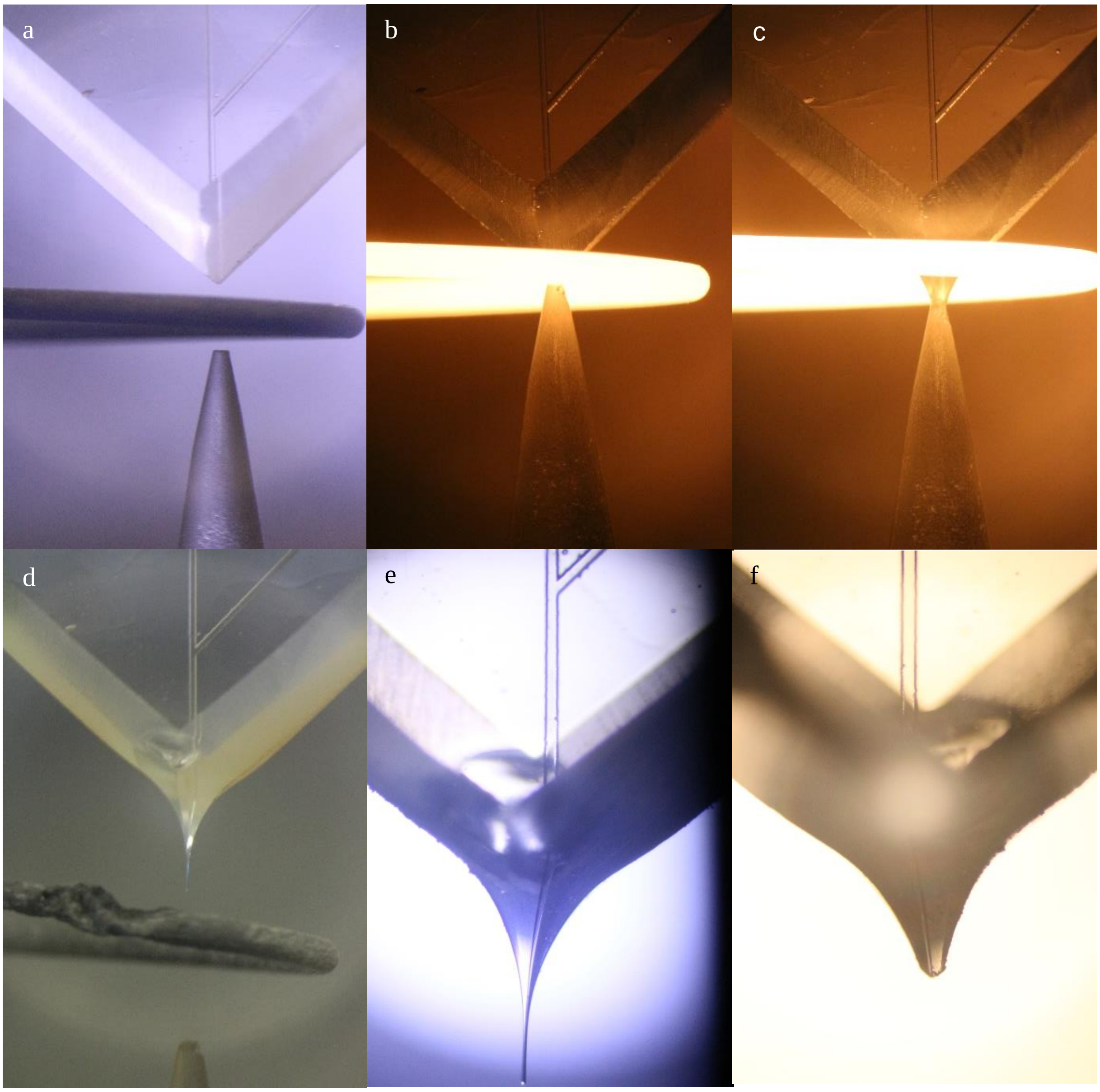

Fig. S3 Images of the pulling process for emitter manufacture (4x magnification). a) Opened chip, metal wire and glass cone. b) Melting of glass cone onto the opened chip. c) Heating of chip tip to elongate glass. d) and e) pulled, and non-opened emitter. f) Opened emitter.

4. Setup for strobe photography and images of desorption process

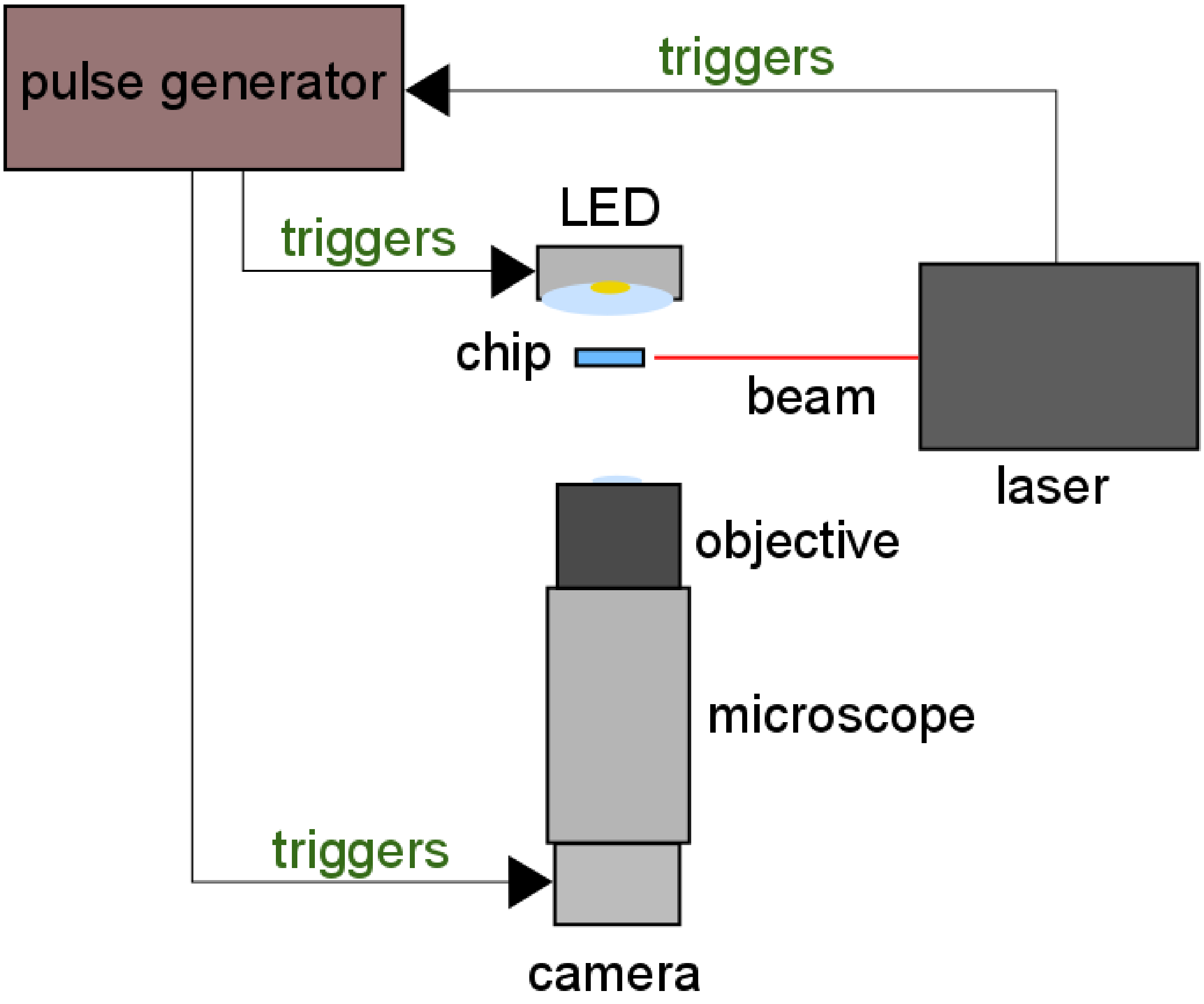

Fig. S4 Scheme of the stroboscope setup, view from above. The chip emits a liquid jet downwards. The focus of the microscope is directed at the liquid jet. To take the images, the laser sends a trigger signal to a pulse generator, which after a certain time triggers a light emitting diode (LED) and a camera. The time is set so that an image of the subsequent laser bombardment is recorded

For the shadowgraphy, a laser shot triggered a signal to the pulse generator. The pulse generator then delayed the signal for LED and camera for a certain time to take a snapshot of the subsequent laser exposure according to equation (1), where t_0 is the time for the initial pulse from the laser, f the frequency of the laser, and t_x the desired time after laser exposure.

$$t_{Snapshot} = t_0 + \frac{1}{f} + t_x \quad (5)$$

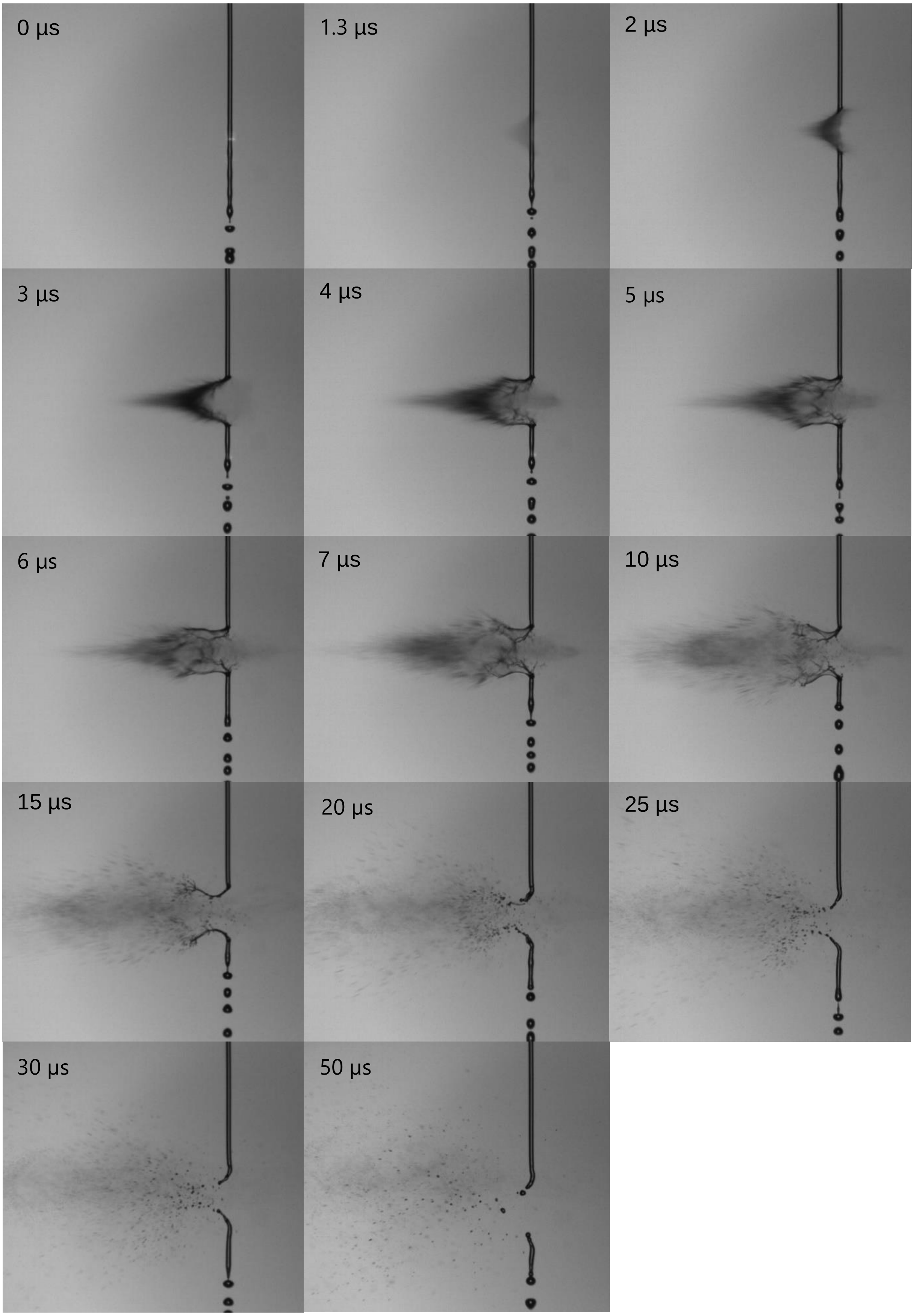

Fig. S5 Shadowgraphy images (time intervals of 100 ns, 10x magnification) of liquid beam 0 µs; 1.3 µs; 2 µs; 3 µs; 4 µs; 5 µs, 6 µs; 7 µs; 10 µs; 15 µs; 20 µs; 25 µs; 30 µs and 50 µs after IR-laser exposure.

5. Survey of dimensions in liquid beam

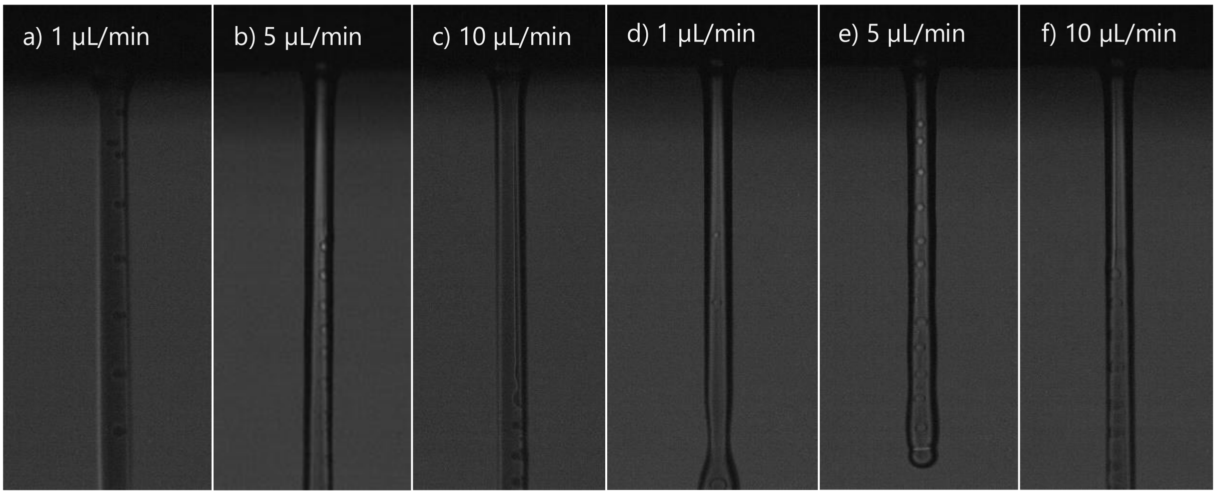

Fig. S6 Shadowgraphy measurements of two-phasic liquid beam consisting of water and from a) to c) of $CH_2Cl_2$and from d) to f) of *n*-heptane. Flow rate of organic liquids was 1 µL/min, 5 µL/min and 10 µL/min, whereas the flowrate of water was 120 µL/min.

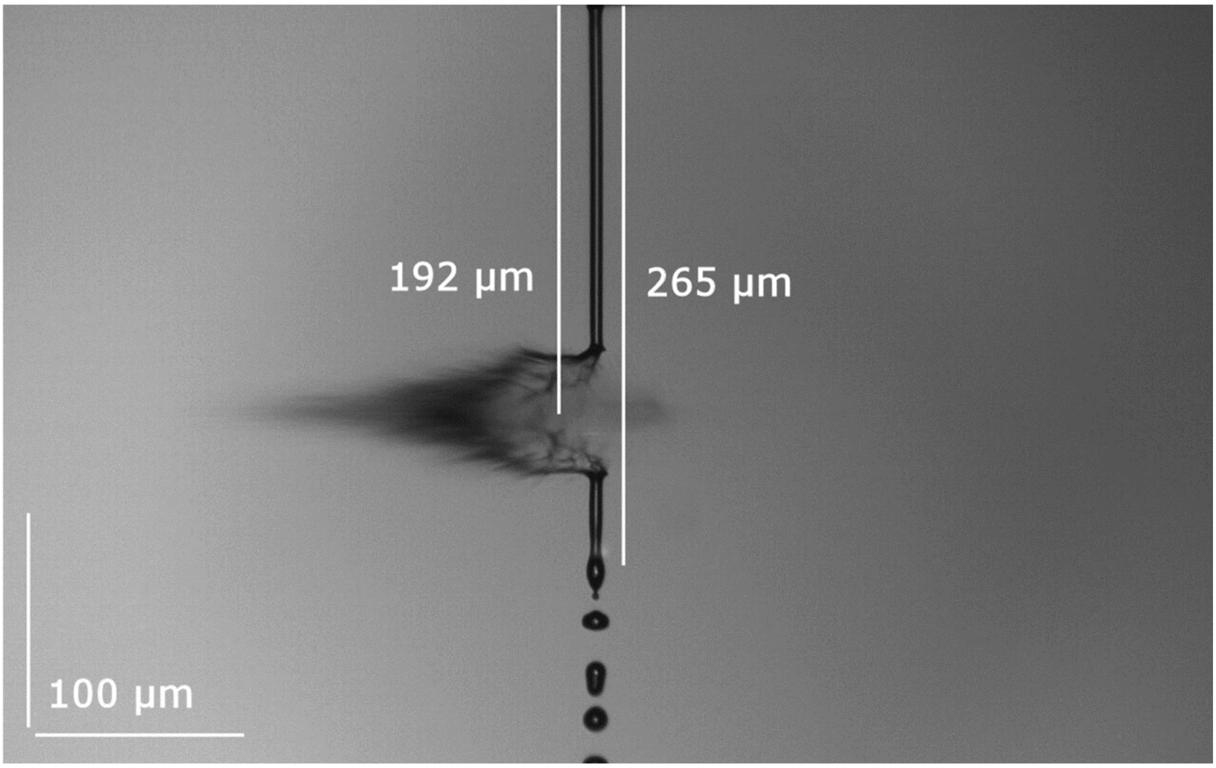

Fig. S7 Shadowgraphy image of liquid beam at 3 µs after laser exposure.

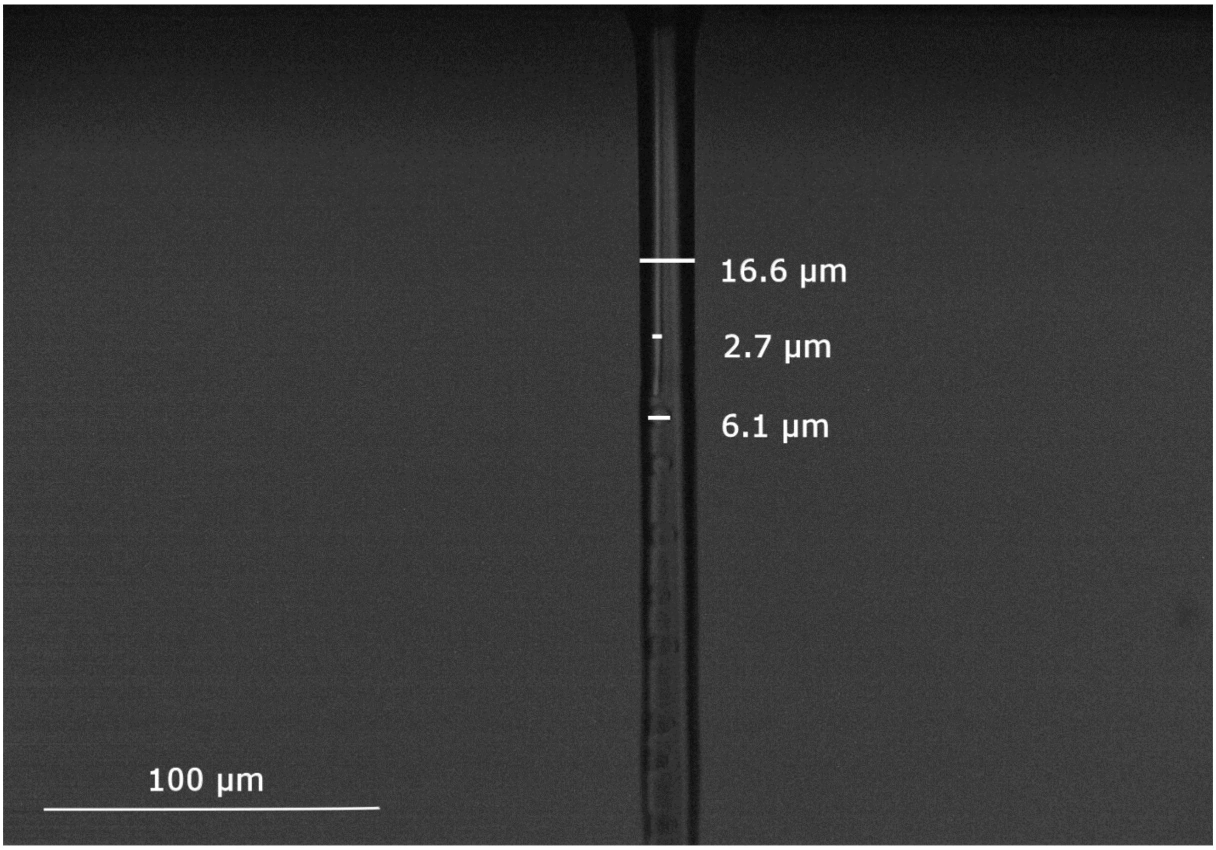

Fig. S8 Shadowgraphy image (20x magnification) of two-phasic liquid beam. Flow rates were u_3=120 µL/min water and u_1=10 µL/min *n*-heptane.

To determine the physical dimensions of the liquid beam, the size of the image in Figure 8 was calculated by multiplying the side length of each pixel with the pixel number and then dividing the length by the magnification of the objective:

Image width: $1600 \cdot 4.5\ \mu m = 7.20\ mm => 7.2\ mm/20\ = 360\ \mu m$ (6)

Image height: $1200 \cdot 4.5\ \mu m = 5.76\ mm => 5.76\ mm/20\ = 288\ \mu m$ (7)

Thus, the size of one image is 360 µ*m* · 288 µ*m*. The diameter for the liquid beam in air, organic phase jet and droplet was calculated similarly.

By dividing the volume of the solution between make-up flow intersection and the point where ionization occurs by the flow rate, the distance between the two positions can be calculated.

Volume from make-up flow to tip: $V(muf - tip) = 0.02\ mm \cdot 0.08\ mm \cdot 10\ mm = 0.016\ mm^3$ (8)

Volume from tip to ionization: $V(tip - iz) = \left(\frac{16.6\ \mu\text{m}}{2}\right)^2 \cdot \pi \cdot 0.192\ mm \approx 4{,}2 \cdot 10^{-5}\ mm^3$ (9)

Volume from make-up flow to ionization: $V_{tot} = \sum V_i \approx 0{,}016\ mm^3$ (10)

To cross the distance from the make-up flow junction to the ionization region, with a set flow rate of $120 \frac{\mu L}{min}$, the solvent needs about 1.6 milliseconds: $t = \frac{V}{u} = \frac{0.016 \cdot 10^{-3}\ \mu L}{120 \frac{\mu L}{min}} \approx 8\ ms$ (11)

6. Droplet frequency calculation:

By comparing the two stroboscope images, the first taken 3 µs after exposure, the second 50 µs after exposure, the flow velocity can be calculated:

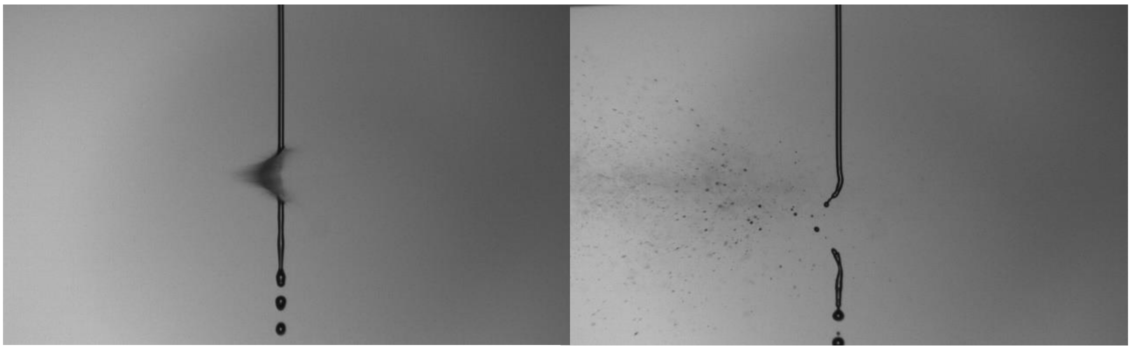

Fig. S9 a) Liquid beam 3 µs after laser exposure. b) 50 µs after laser exposure

It can be seen, that the beam moves a little bit downwards in this time of 47 µs. Using the rule of three you can calculate how long it takes to replace the complete beam (about 260 µs).

$$\frac{2{,}5\ cm}{47\ \mu s} = \frac{14\ cm}{x}$$

$$x \approx 260\ \mu s$$

By comparing the flow rate to the number of droplets in the beam in the next image, approximately 10, it can be calculated that about 38000 drops are generated per second. This is a rough estimation, since the droplet frequency depends on the ratio of used solvents, and the capillary number C_a. Also, the droplet images were shot with an objective with different magnification. Flow rates in this image were 5 µL/min n-heptane, 120 µL/min water.

$$f = \frac{10\ droplets}{260\ \mu s} = 38461 \frac{droplets}{s} \approx 38000 \frac{droplets}{s}$$

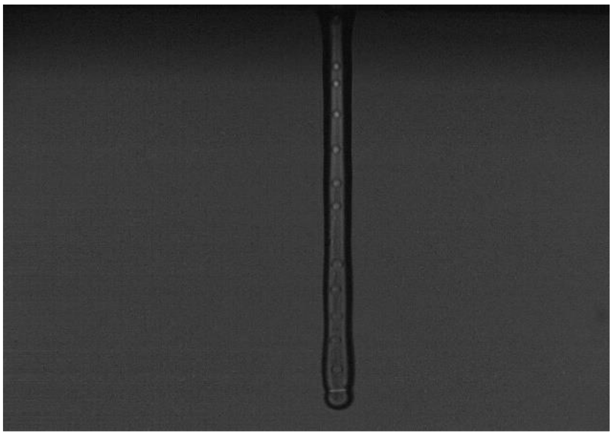

Fig. S10 Aqueous liquid beam with n-heptane droplets with the flow rates 5 µL *n*-heptane, 120 µL/min water.

7. IR-MALDI mass spectra of polyaromatic hydrocarbons

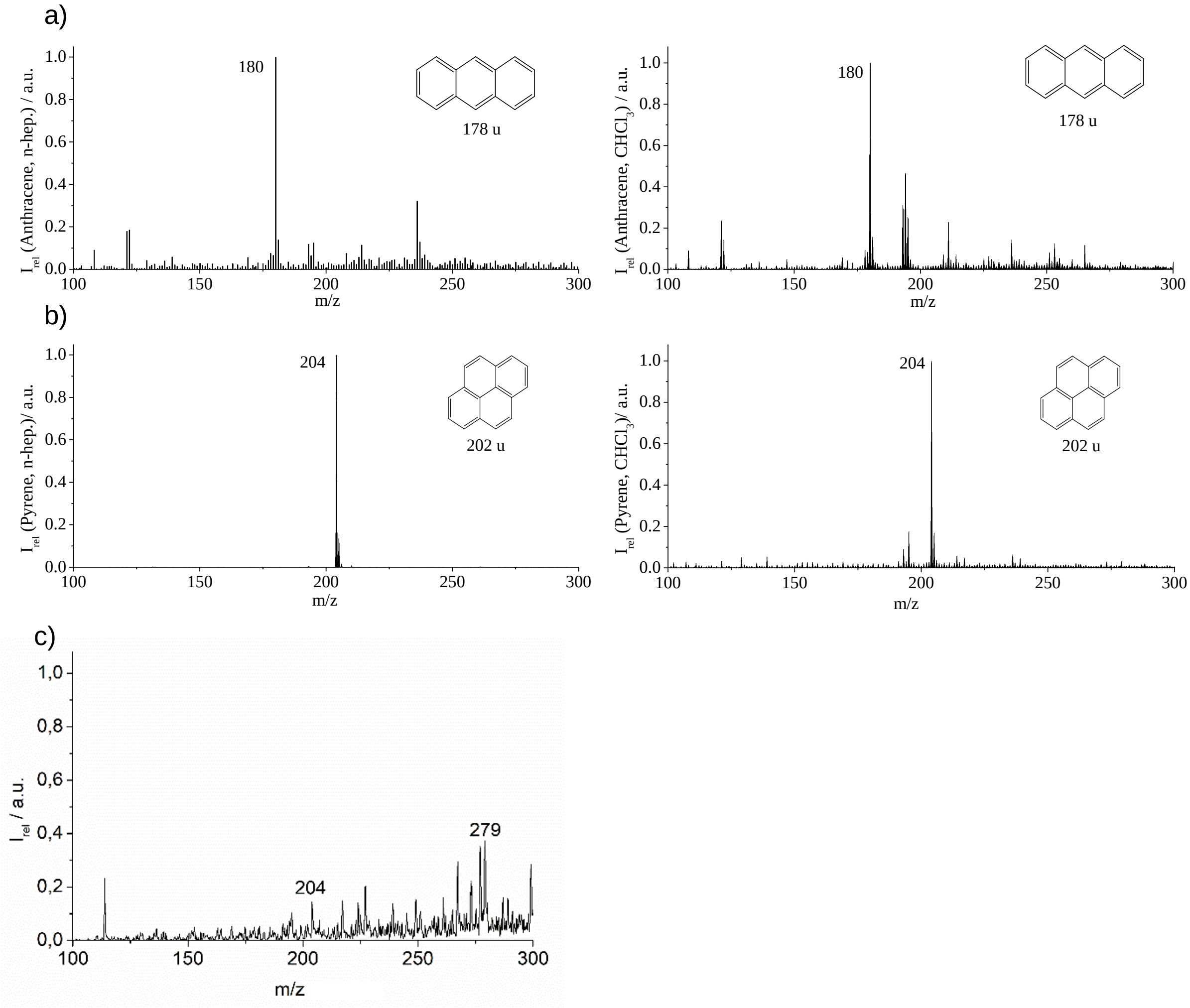

Fig. S11 IR-MALDI-Mass spectra of 1 mg/mL solutions in *n*-heptane (left side) and chloroform (right side). a) anthracene and b) pyrene (flow rates: 5 µL/min solution, 115 µL/min matrix). c) Saturated pyrene solution in water (flow rate: 120 µL/min aqueous solution). The signal response at m/z 204 is clearly lower compared to the signal from the organic solutions, even though the sample doesn't get diluted by matrix.

8. Chip-IR-MALDI-MS concentration measurements: Caffeine dissolved in $CHCl_3$ and LOD calculation

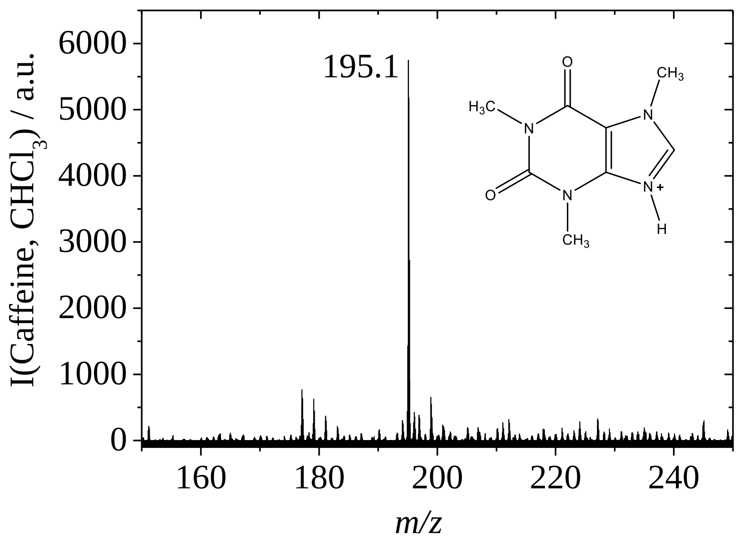

Fig. S12 Chip-IR-MALDI-MS mass spectra of 50 µM caffeine in $CHCl_3$.

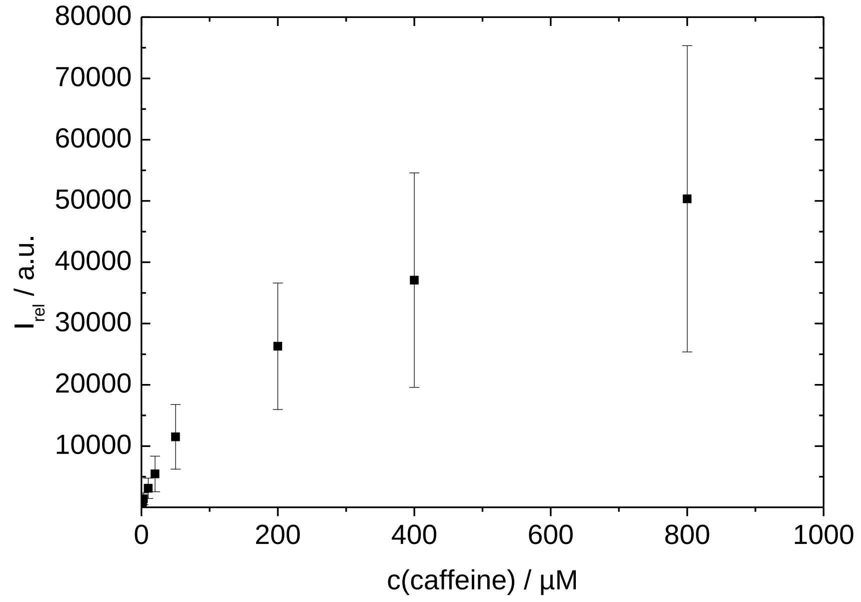

Fig. S13 Chip-IR-MALDI-MS measurements (n = 3) of different concentrations of caffeine in $CHCl_3$. Flow rates: u_1(solution) = 5 µL/min, u_3(water) = 115 µL/min. The signal of protonated caffeine at 195 m/z shows a linear response up to a concentration of 50 µM, and then saturation occurs.

c(caffeine) / µM	I_{avg} / a.u.	Std / a.u.	Linear regression	
0	551	65	t	m
1,5	840	311	725,5474	219,2037
3	1395	975		
10	3122	1653		
20	5455	2910		
50	11512	5268		
200	26289	10315		
400	37080	17509		
800	50354	24983		

The function for linear regression of the graph between 0 and 50 µg/mL is

$$y = 219.2\frac{1}{\mu M}x + 725.55 \quad (3)$$

By converting (1) and inserting y=0, the signal to noise ratio S/N can be determined:

$$x = \frac{y-725.22}{219.2\frac{1}{\mu M}};$$

$$LOD = 3\frac{S}{N} = \frac{(3*551)-725.22}{219.2\frac{1}{\mu M}} \approx 4.3\ \mu M$$

9. Photochemical oxidation of *N*-phenyl-1,2,3,4-tetrahydroisoquinoline

For the photochemical oxidation of *N*-phenyl-1,2,3,4-tetrahydroisoquinoline the chip was irradiated with twelve OSRAM Oslon SSL royal blue (455 nm) attached to an aluminium heat sink. The LEDs were operated at 700 mA. Substrate was synthesized by the following proceedure.

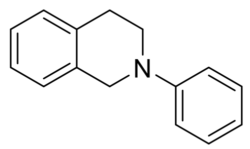

$C_{15}H_{15}N$
209.29 g/mol

In an ovenwarm Schlenk flask 95.2 mg (0.50 mmol, 10 mol %) CuI, 1.96 g (9.25 mmol, 1.85 equiv) K_3PO_4 and 0.97 g (7.25 mmol, 1.45 equiv) 1,2,3,4-tetrahydroisoquinoline were suspended in 5.5 mL 1-propanol/ethylene glycol 10:1 (*v*/*v*). Finally, 1.02 g (5 mmol, 1.0 equiv) iodobenzene were added and the reaction mixture was stirred at 95 °C for 24 h. 20 mL Et_2O and 20 mL H_2O were added and the aqueous phase was extracted with Et_2O (3x 20 mL). The combined organic phases were dried over Na_2SO_4 and the solvent was removed under reduced pressure. The crude produtct was purified by flash chromatography on silica gel (petroleum ether/ethyl acetate, 3-15% ethyl acetate) to afford 658 mg (3.14 mmol, 63%) of the title compound as a colourless solid.

Retention factor: R_f = 0.30 (petroleum ether/ethyl acetate 95:5 *v*/*v*)

^{1}H NMR (400 Mhz, $CDCl_3$, 25 °C): δ = 7.35 - 7.24 (m, 2H), 7.23 - 7.11 (m, 4H), 7.01 (d, *J* = 8.0 Hz, 2H), 6.85 (t, *J* = 7.5 Hz, 1H), 4.43 (s, 2H), 3.58 (t, *J* = 6.0 Hz, 2H), 3.00 (t, *J* = 6.0 Hz, 2H)).

^{13}C NMR (100 Mhz, $CDCl_3$, 25 °C): δ = 150.6, 135.0, 134.6, 129.3, 128.7, 126.7, 126.5, 126.2, 118.8, 115.3, 50.9, 46.7, 29.2.

HR MS (ESI+, MeCN): m/z = 210.1277 $[M+H]^+$, calc.: 210.1277.

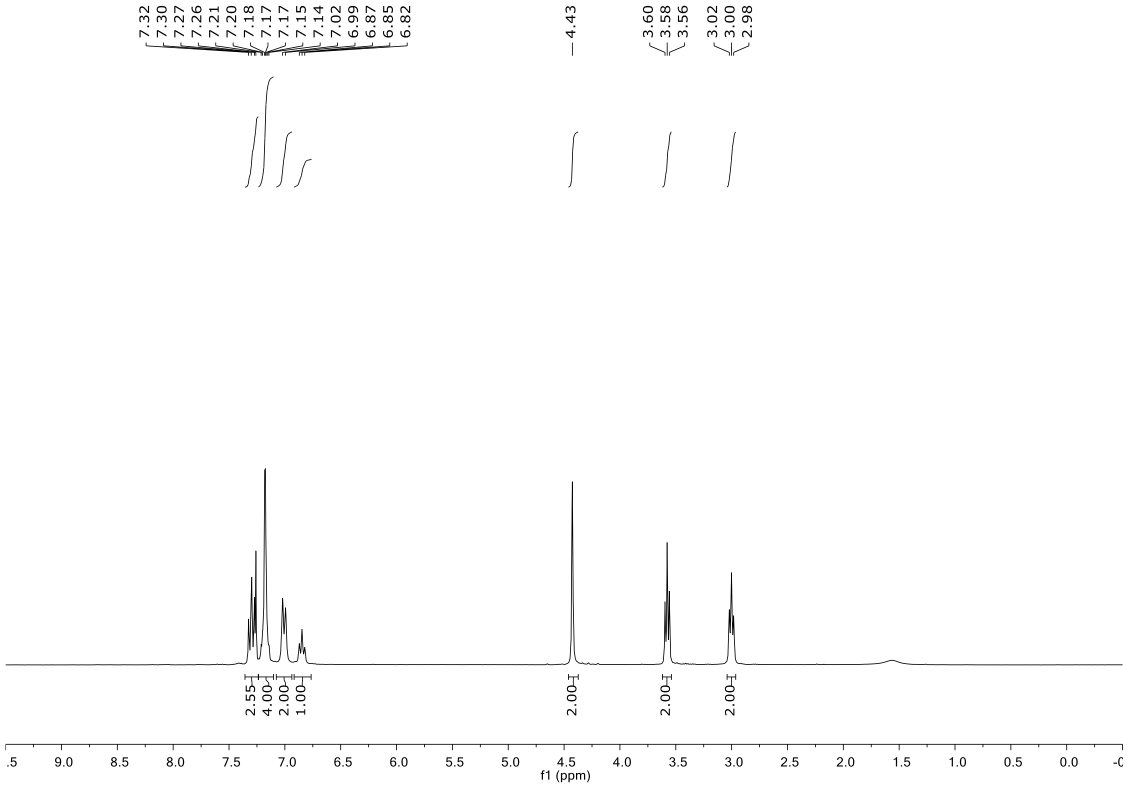

Fig. S14 ^{1}H NMR spectrum of *N*-phenyl-1,2,3,4-tetrahydroisoquinoline in $CHCl_3$ applied for on-chip photooxidation experiments.

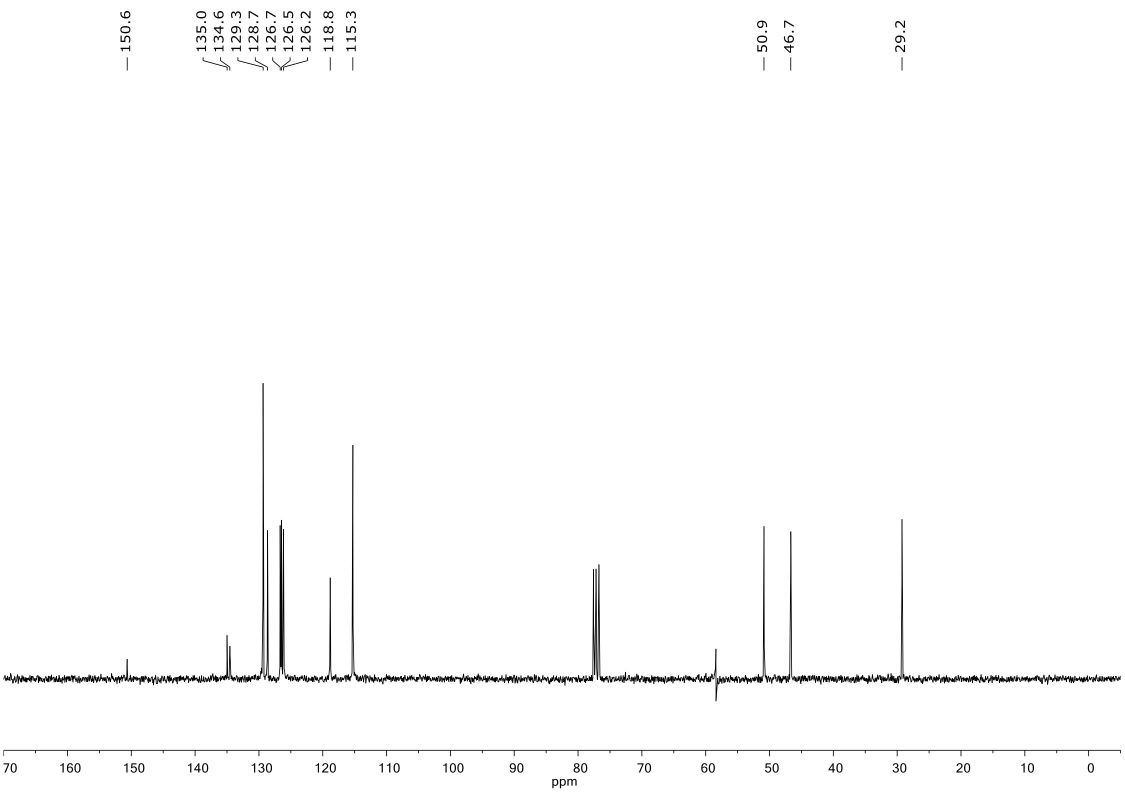

Fig. S15 ^{13}C NMR spectrum of *N*-phenyl-1,2,3,4-tetrahydroisoquinoline in $CHCl_3$ applied for on-chip photooxidation experiments.

References

[1] E. Heumann, *Zeitschrift für Naturforschung A* **1981**, *36*.

[2] K. E. Gustafson, R. M. Dickhut, *J. Chem. Eng. Data* **1994**, *39*, 281–285.

[3] D. Leaist, L. Hui, *J. Phys. Chem.* **1990**, *94*, 8741–8744.

[4] Thürmann, Sebastian: *HPLC in Glaschips*, Dissertation, Leipzig, **2015**.
